# Colorectal travelling fellowships: Exploring current perspective and future direction

**DOI:** 10.1016/j.sopen.2023.07.005

**Published:** 2023-07-20

**Authors:** Ahmed H. Badrek-Amoudi

**Affiliations:** Faculty of Medicine, Umm Al-Qura University, Saudi Arabia

**Keywords:** Surgical travelling fellowship, Colorectal travelling fellowship, Surgical fellowship, Travel grant, Travel scholarship, Covid-10 pandemic

## Abstract

Travelling Surgical fellowships (TSF) have a longstanding tradition in promoting out-of-programme surgical training, fostering collegiality and collaboration among surgeons. In this retrospective review we explore its historical context and examine existing practices and likely future trends. More specifically, we focus on colorectal travelling fellowships (CTF) and provide additional quantitative and qualitative analyses, highlighting the most valued theme-based surgical experiences and examine their merits and impacts. The TSF time-series analysis was based on a total of 350 awarded fellowships from 2000 to 2019. CTF analysis was based on 98 fellowships. The accelerated utilization of internet-based virtual interaction during the COVID-19 Pandemic has offered an opportunity to examine its possible intermediate and long-term disruptive effects.

## Highlights

• This article provides a review and analysis of trends, values, and impacts of surgical travelling fellowships (TSF) and colorectal travelling fellowships (CTF).
• The article discusses the incentives for pursuing a travelling fellowship, such as gaining technical surgical knowledge, advancing one's career, presenting research, and connecting with expert surgeons.
• The most sought-after surgical themes in CTF were identified as minimally invasive surgery, the organization of the host healthcare, surgical training and mentoring, multidisciplinary management of challenging colorectal cases, and new approaches to pelvic floor and proctological conditions. The article also discusses the impact of travelling fellowships on surgeons from developing countries, particularly on their attitude and institutional work-flow in managing complex colorectal cases, as well as community-based initiatives such as screening programs.
• The article concludes with a discussion of the potential impact of the COVID-19 pandemic on travelling fellowships and the adoption of new technologies facilitating virtual interaction

## Background

Travel has long been considered a tradition associated with surgical development, learning, and friendship. Its influence can be traced as far back as the early nineteenth century when it helped to uproot established surgical traditions whilst facilitating the democratizations within surgical practice [3,4]. Following World War I (WWI), it provided an impetus for the dissemination of operative knowledge, the use of new technologies, the convergence of surgical practices, and the establishment of professional surgical organizations [1,2].

Facilitated by improved means of transportation and enabled by periods of world peace and prosperity, the concept of Surgical Travelling Fellowships (TSF) has evolved in response to the rapid advances in surgical practice. Early accounts by eminent surgeons such as William Halsted (1904) and Edward Churchill, (1926) both from the United States indicate how travel to multiple academic surgical institutions in Europe had a lasting impact on their patient care and on the subsequent foundation of structured graduate training programs for surgeons in the United States [5]. Other examples include the Travelling Surgical Society of Great Britain and Ireland, established in 1924, and the Moynihan Travelling Fellowship, founded in 1937, both of which recognised the need to promote travel and TSFs to showcase achievements in surgery and to improve surgeon-to-surgeon connectivity, nationally and internationally [6,7].

In colorectal surgery, TSFs (CTF) have benefited more recently from the international partnerships with colleges of surgery, surgical associations, as well as colorectal and philanthropic societies. The strategic aim has been to encourage out-of-programme surgical training, to offer learning and training opportunities to surgeons from middle and low-income countries, and to foster collegiality and collaboration among the international surgical community [10,11,14].

The current pathway for CTS consists of a selection process for candidate, leading to the award of a sponsorship, followed by travel and host-based engagements. The financial support, duration, and program structure vary considerably and are largely determined by the specific mission for each fellowship (Table 1).

**Table 1:** Sponsoring organizations, Duration of TSF programmes, published number of awards and numbers of TSF website reports available.

Sponsoring organisation	From	To	TSF awards	Reports	Duration/funding
American Society of Colon & Rectal Surgery (ASCRS) [10]	2007	2017	52	9^a^	Fair: Travel, Registration
Association of Surgeons of Great Britain and Ireland (ASGBI) [11]:					N/A
Moynihan Travelling Fellowship	1937	2018	49	N/A	
BJS	1979	2019	N/A	N/A	
Colorectal Surgical Society of Australia and New Zealand [12]	N/A	N/A	N/A	N/A	N/A
European Society of COLOPROCTOLOGY (ESCP) [13]	2010	2019	72	72	1–2 weeks Fair: Travel, Registration, Stay
James IV Association of Surgeons (JIVAS) [14]	2002	2019	34	34^b^	
Royal College of Physicians and Surgeons of Canada (RCSC) [15]	2016	2019	110	N/A	N/A
Royal College of Surgeons in Ireland (RCSI) [16]				71^b^	£1000
Section of Coloproctology, Royal Society of medicine	2001	2020	20		
Colle Travelling Fellowship in Surgery	1985	2020	50		
Ethicon Foundation Travel Grants	2009	2020	83		
Dr. Richard Stevens Scholarship	2013	2019	6		
Royal College of Surgeons of Edinburgh (RCSEd) [17]:	N/A	N/A	N/A	N/A	N/A
Tuanku Muhriz Fellowship Rural Surgery					
Athe Alban Barrod D'Sa Memorial travelling Fellowship In G.S					
Sir James Fraser Travelling Fellowship in General Surgery					
Royal College of Surgeons of England (RCSE) [18]	2016	2020	N/A	N/A	N/A
The Association of Coloproctology of Great Britain and Ireland (ACPGBI) Travelling Fellowships to Australia, Europe and USA [19].	2015	2018	N/A	5	N/A
The Society for Surgery of the Alimentary Tract (SSAT) [20]	2015	2019	10	10^a^, ^c^	$8000, 1–2 weeks
The Society for Surgery of the Alimentary Tract (SSAT) & Indian Association of surgical Gastroenterology (IASG) [21]	2016	2018	3	3^a^, ^c^	$5000, 2 weeks
Travelling Surgical Society of Great Britain and Ireland: Price Thomas Fellowship [7]	2009	2019	N/A	11^c^	N/A

a International Travelling Fellowships.

b Multi-Specialty Travelling Fellowships.

c Non-Colorectal.

Pre-Covid figures on the number of CTF, published by the European Society of Coloproctology (ESCP), show a sustained increase in supply and demand [13]. However, and despite this up trend, there has been limited published evidence to support its added value or lasting impact. On the other hand, the creative use of online platforms in surgical education, collaboration, and training [8,9,[22], [23], [24], [25]], is now increasingly been offered as an alternate to traditional face-to-face activities. This includes live surgeries, continuous surgical education, attending conference, and surgical mentoring.

Within this context, the value of travel and physical face-to-face engagements needs to be explored and defined. The aim of this paper is to address this need by examining the current trends of TSF/CTF and their future direction, in addition to providing analysis on the most valued experiences within CTF, as well as the overall professional and personal impacts.

## Method

### Search terms, inclusion and exclusion criteria

Using Google and Google Scholar search engines, the following terms were searched:

Surgical/ surgery travelling/ travelling fellowship,

Colorectal travelling/ travel/ fellowship

Surgical/surgery travel grant,

Surgical/ surgery travel scholarship.

**Diagram 1:**
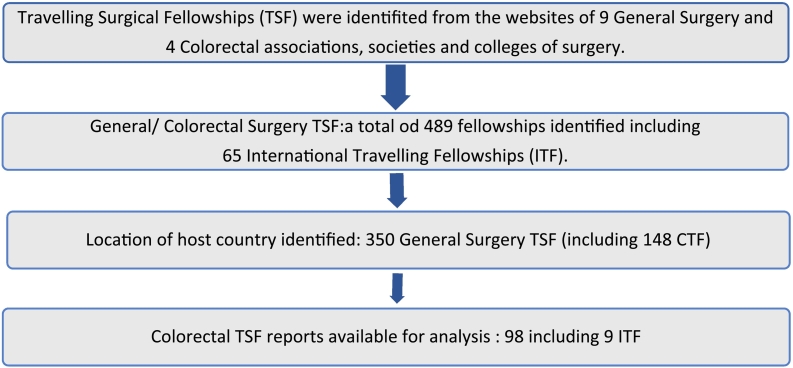
Process: identifications of STF, ITF and Colorectal STF.

We conducted a retrospective review of all TSFs, using the search terms listed above, limited to publications in the English language. Specialties outside the General Surgery domain (e.g., cardiac, plastic and orthopaedics) were excluded. When available, online Colorectal TSF (CTF) reports were identified and independently processed and analysed by two senior surgeons (Table 1). The identification process is summarized in diagram 1.

General Surgical subspecialties (e.g., Breast and Endocrine, Hepatobiliary, and Vascular surgeries) were included in trend and time-series analysis.

### TSF data processing

For each TSF, a record was made of the host country and the year in which TSF was awarded. Countries were then grouped into nine geo-specific regions as shown in Table 3. In this data set we have performed a time series analysis, detailed in the following section (Primary data analysis).

### Data analysis

To account for any presumed serial correlation between years, we utilized autoregressive integrated moving average (ARIMA) time series analysis. Autoregression (AR) is the term referred to the period of autocorrelation. In our data set, the unit measure was 1 year, defined as the year in which TSF was awarded. The ARIMA and AR terms were identified using autocorrelation and partial autocorrelation graphs.

A time series dataset must be determined as stationary and non-random to allow effective prediction. The model's suitability was checked using the Augmented Dickey-Fuller (ADF) test. Goodness of fit was assessed by r^2^ statistics.

A comparison between the total and annual percentages for each theme category was determined using chi-square test, a *p*-value < 0.05 was considered significant.

Data analysis was achieved using EViews 9 and IBM SPSS 25 for Windows X.

### Colorectal TSF analysis

Web-based CTF reports identified were analysed. In each, surgical experiences were distilled into ten theme categories (TC), outlined in Table 2. A record was made for each TC based on intended, observed, assisted, or performed experiences mentioned in the reports. The annual frequency and distribution of host countries were determined (sentence deleted). A further analysis of the data set from 2010 to 2019 was performed to show the annual and total percentage share for each theme-category. Statistical comparison between theme categories was conducted.

**Table 2:** Themes and rationale distilled from travelling surgical fellowship report.

	Theme	Rationale
1	ROBOTIC SURGERY	Colorectal resections, TME and CME
2	TaTME & TAMIS SURGERY	Transanal resections and mucosal excisions
3	RESEARCH	Research discussions, participation and future collaborations
4	PELVIC FLOOR & FUNCTIONAL DBOWEL DISORDERS	Clinical, physiological and radiological assessments.
		Surgeries including STARR, VMR, Transanal approaches, SNS and magnetic beads
5	ORGANISATION TRAINING & EDUCATION	Structural organisation, perioperative care.
		Health care funding, working hours.
		MDT meetings.
		Surgical resident/ fellow mentorship and training.
		Educational activities.
6	HIPEC, LATERAL PELVIC WALL LN DISSECTION & PELVIC EXENTRATION (HLP)	Radical surgeries.
7	LAPAROSCOPY & SILS	Laparoscopic and single or minimal port access colorectal resection,
8	IBD, INTESTINAL FAILURE	Diagnosis, support surgeries including stoma formation, pouch surgery and small bowel transplantations surgeries
9	HAEMORRHOIDS & FISTULA SURGERIES	Including PPH, mucocutaneous flaps, LIFT and other procedures
10	CONFERENCE, WORKSHOPS, MASTER CALSSES	Attendance, mentors and speakers

The qualitative descriptive analysis consists of a summary of general motives, impressions, and impacts was recorded.

### International Colorectal Travelling Fellowship (ICTF)

A separate descriptive analysis was also performed on CTSs awarded to surgeons from middle- and low-income countries.

TME: Total Meso-rectal Excision, CME: Complete Meso-colic Excision, STARR: Stapled Trans anal Rectal Resection, VMR: Ventral Mesh Rectopexy, MDT: Multi-Disciplinary Team, PPH: Procedure for Prolapse and Haemorrhoids., LIFT: Ligation of Inter-sphincteric Fistula Tract.

## Results

### Global distribution, annual frequency general surgery TSF

**Diagram 2:**
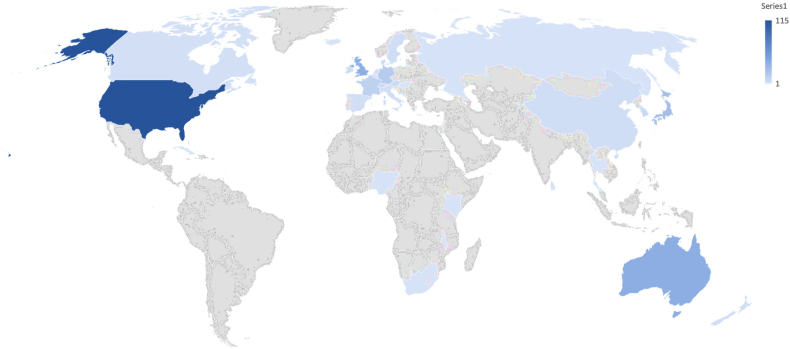
World map showing the relative global share of travelling surgical fellowship as host countries.

**Graph 1:**
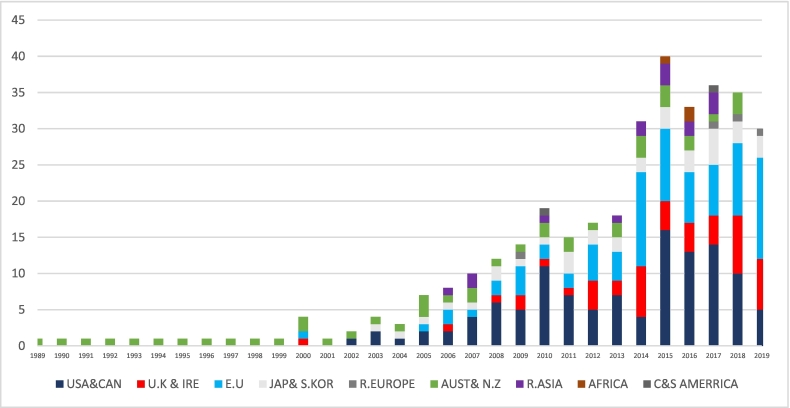
A stacked column graph showing 20-year annual distribution of sponsored TSF and their host countries.

Online search yielded 489 General Surgery TSFs including 98 Colorectal TSF (CTF). Information regarding host countries were identified in 350 TSFs. The results are detailed in Table 3, Diagram 2, and Graph 1. The majority of TSFs identified between 1989 and 2000 were hosted by Australia. Subsequent (post-2000) worldwide distribution of host countries shows the following: North America accounting for 33 %, The European Union 24 %, The United Kingdom 13 %, Australia 12 %, Japan and South Korea accounting for 10 %. An increase in the number of fellows is demonstrated from 2000 onwards. A slight decline in overall numbers is noted in 2017–19 and is primarily attributed to a decline in the number of international fellows to the United States.

**Table 3:** Annual distribution of number of sponsored TSF in grouped countries and unions.

	USA&CAN	U.K&IRE	EU	JAP& S.KOR	R. EUROPE	AUST& NZ	R.ASIA	AFRICA	C&S AMERRICA	TOTAL/ANUM
1989	0	0	0	0	0	1	0	0	0	1
1990	0	0	0	0	0	1	0	0	0	1
1991	0	0	0	0	0	1	0	0	0	1
1992	0	0	0	0	0	1	0	0	0	1
1993	0	0	0	0	0	1	0	0	0	1
1994	0	0	0	0	0	1	0	0	0	1
1995	0	0	0	0	0	1	0	0	0	1
1996	0	0	0	0	0	1	0	0	0	1
1997	0	0	0	0	0	1	0	0	0	1
1998	0	0	0	0	0	1	0	0	0	1
1999	0		0	0	0	1	0	0	0	1
2000	0	1	1	0	0	2	0	0	0	4
2001	0	0	0	0	0	1	0	0	0	1
2002	1	0	0	0	0	1	0	0	0	2
2003	2	0	0	1	0	1	0	0	0	4
2004	1	0	0	1	0	1	0	0	0	3
2005	2	0	1	1	0	3	0	0	0	7
2006	2	1	2	1	0	1	1	0	0	8
2007	4	0	1	1	0	2	2	0	0	10
2008	6	1	2	2	0	1	0	0	0	12
2009	5	2	4	1	1	1	0	0	0	14
2010	11	1	2	1	0	2	1	0	1	19
2011	7	1	2	3	0	2	0	0	0	15
2012	5	4	5	2	0	1	0	0	0	17
2013	7	2	4	2	0	2	1	0	0	18
2014	4	7	13	2	0	3	2	0	0	31
2015	16	4	10	3	0	3	3	1	0	40
2016	13	4	7	3	0	2	2	2	0	33
2017	14	4	7	5	1	1	3	0	1	36
2018	10	8	10	3	1	3	0	0	0	35
2019	5	7	14	3	1	0	0	0	0	30
TOTAL NUMBER OF FELLOWS										350

USA & CAN: United States of America and Canada, UK & IRE: United Kingdom and The Republic of Ireland, EU European Union, JAP & S.KOR: Japan and South Korea, R. Europe: rest of Europe, Aust & NZ: Australia and New Zealand, R. Asia: Rest of Asia, C&S America: Central and South America.

### Time series analysis

Our time-series data proved non-stationary by ADF test (t-statistics −2.087839, probability 0.5314) necessitating transformation before analysis. Successful transformation was achieved using the Auto-Regressive Integrated Moving Average (ARIMA) model of (1,1,0). AR(1) indicating autocorrelation between the total number of fellows in the previous year with the number of fellows in the current year, I1 indicating a difference of (1) and a moving average (MA (0)) of 0, ADF test (t-statistics −4.339066, probability 0.0113). The time series analysis is shown in Table 4. A regression coefficient (R^2^) of 0.257, shows a year-on-year weak positive correlation. It implies that time alone accounts for 26 % of the variability seen with a significant *p*-value of 0.018 (F-statistics).

**Table 4:** ARIMA^a^ model (1,1,0) time series analysis.

Variable	Coefficient	t-Statistic	Probability
C	0.117670	1.413277	0.1690
AR(1)	−0.494668	−2.827077	0.0087
SIGMASQ	0.147150	3.366407	0.0023
R-squared	0.257059		
Adjusted R-squared	0.202027		
S.E. of regression	0.404351		
Sum squared resid.	4.414494		
Log likelihood	−13.96391		
F-statistic	4.671033		
Prob(F-statistic)	0.018108		
Inverted AR Roots	−0.49		

a ARIMA: Auto-Regressive Integrated Moving Average.

## International Colorectal Travelling Fellowships (ICTF)

The cohort of international fellows identified in this study were awarded by ASCRS and SSAT for visiting the USA (Table 1). 65 International Travelling Fellowships were awarded between 2007 and 2019, only nine colorectal ITF reports are currently available. Fellows were invited to attend the ASCRS Annual Scientific Meeting and then offered observership programs at centres of excellence in the United States. A descriptive summary of the reported impacts and limitations by international fellows is provided in Table 5.

**Table 5:** Impact of ITF on individual fellows and limitations in home country.

Impact	Limitation
1. Start joint research projects	1. Funding and institutional organisation
3. Establish journal clubs and evidence-based practice	2. Human and technical resource limitations
4. Development of specialist colorectal surgery training program	3. Case volume
5. Professional development programmes for doctors and nurses	
6. Improvements in surgical techniques: ▪ Pouch surgery ▪ Pelvic floor rectal prolapse ▪ Use of perineal blocks in ambulatory rectal procedures ▪ Management of anastomotic leaks ▪ Management of Crohn's Disease ▪ Multidisciplinary colorectal cancer management	
7. Initiate plan to develop advanced laparoscopic and minimally invasive surgery	
8. Enhanced Recovery protocols After Surgery	
9. Effort to start colorectal cancer screening programme	
10. Commencement of a multi-disciplinary team management in colorectal cancer	

## Colorectal TSF host countries

148 Colorectal TSF visits to a total of 19 countries were identified. A breakdown of the host countries is shown in Table 6 & Graph 2. Ninety-eight reports, were sourced from ESCP (72), ASCRS (9), ACPGBI (5), James IV AS (3), and RCSI (9). Many organizations, including ACS, ASGBI, RCS(Ed), RCS(Eng), and RCSC with known CTF programs, have not provided online information. The figures for the USA were primarily based on international TSFs. Outside the USA, the top five countries hosting CTF were the U.K., France, Japan, South Korea and Switzerland. The average number of participating hospital-centres in each country is three (range 1–8).

**Table 6 & Graph 2:**
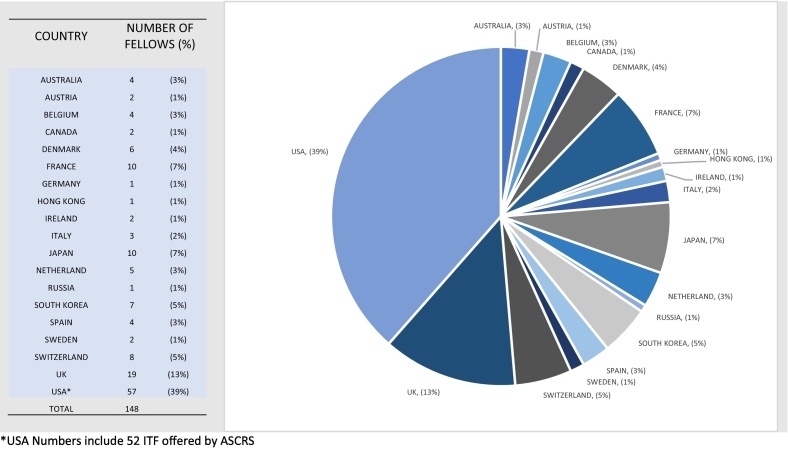
Host countries and number of TSF offered since 2010.

^⁎^USA Numbers include 52 ITF offered by ASCRS.

## Data analysis of colorectal theme-categories

**Graph 3:**
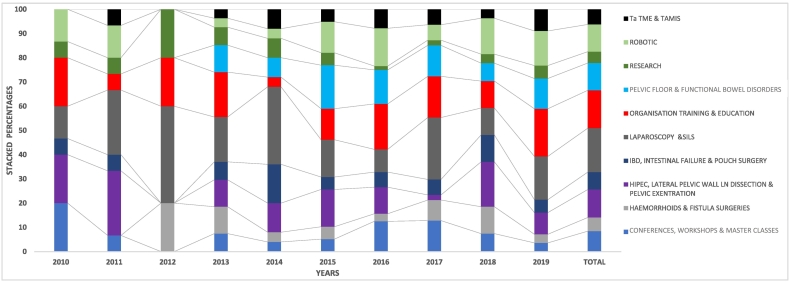
A 100 % Stocked Column Graph demonstrating annual and total percentage distribution of CTF themes-Categories.

The findings are outlined in Table 7 and Graph 3. Ninety-eight colorectal TSFs reports (CTF) were examined, yielding 320 reported theme experiences. The total cumulative difference between theme categories was shown to be significant (X^2^ = 48.96, p-value <0.001). The top five theme preferences were, laparoscopy at 18 % (9–40), organisation, education and training at 16 % (4–20), radical surgeries represented by HPL at 12 % (2–27), robotic surgery, pelvic floor and functional bowel disorders, both themes at 11 % (0–18). Time series analysis for each theme category was not possible due to the limited number of observed experiences for each category. The periods of surgical techniques emergence appear to correlate with the year candidates expressed interest. Examples include TaTME in 2011, Ligation of Inter-sphincteric Fistula tract in 2012 and in Ventral Mesh Rectopexy in 2013.

**Table 7:** 10-year distribution of Colorectal TSF themes.

	2010		2011		2012		2013		2014		2015		2016		2017		2018		2019		TOTAL	
	N	%	N	%	N	%	N	%	N	%	N	%	N	%	N	%	N	%	N	%	N	%
1. CONFERENCE, WORKSHOPS, MASTER CLASSES	3	20	1	6.7	0	0	2	7.4	1	4	2	5.1	8	13	6	13	2	7.4	2	3.6	27	8
2. HAEMORRHOIDS & FISTULA SURGERIES	0	0	0	0	1	20	3	11	1	4	2	5.1	2	3.1	4	8.5	3	11	2	3.6	18	6
3. HIPEC, LATERAL PELVIC WALL LN DISSECTION & PELVIC EXENTRATION (HPL)	3	20	4	27	0	0	3	11	3	12	6	15	7	11	1	2.1	5	19	5	8.9	37	12
4. IBD, INTESTINAL FAILURE & POUCH SURGERY	1	6.7	1	6.7	0	0	2	7.4	4	16	2	5.1	4	6.3	3	6.4	3	11	3	5.4	23	7
5. LAPAROSCOPY & SILS	2	13	4	27	2	40	5	19	8	32	6	15	6	9.4	12	26	3	11	10	18	58	18
6. ORGANISATION TRAINING & EDUCATION	3	20	1	6.7	1	20	5	19	1	4	5	13	12	19	8	17	3	11	11	20	50	16
7. PELVIC FLOOR & FUNCTIONAL BOWEL DISORDERS	0	0	0	0	0	0	3	11	2	8	7	18	9	14	6	13	2	7.4	7	13	36	11
8. RESEARCH	1	6.7	1	6.7	1	20	2	7.4	2	8	2	5.1	1	1.6	1	2.1	1	3.7	3	5.4	15	5
9. ROBOTIC SURGERY	2	13	2	13	0	0	1	3.7	1	4	5	13	10	16	3	6.4	4	15	8	14	36	11
10. Ta TME & TAMIS	0	0	1	6.7	0	0	1	3.7	2	8	2	5.1	5	7.8	3	6.4	1	3.7	5	8.9	20	6
TOTAL NUMBER OF THEMES EXPERIENCES IDENTIFIED	15	100	15	100	5	100	27	100	25	100	39	100	64	100	47	100	27	100	56	100	320	100
CHI SQUARE (X^2^)	17.214		5.222		22.149		17.250		9.974		17.000		6.704		0.600		6.867		2.267		48.960	
P-value	0.045		0.815		0.008		0.045		0.353		0.049		0.668		0.896		0.443		0.894		0.000

## Descriptive analysis of colorectal theme-categories

Distilled theme categories reveal a broad distribution of multiple interests. Recurring theme-country associations are noted. Examples include: interest in lateral pelvic wall dissection observed in Japan, robotic surgery in South Korea, assessment and management of functional bowel disorders in the UK, pelvic floor dysfunction and ventral mesh rectopexy in Belgium, LIFT and advancement flaps in the Netherlands, PPH, STARR, and Trans-STARR in Italy, Transanal TME in Spain and advanced surgical approach to IBD in the USA.

With reference to minimally invasive surgery, fellows reported new insights into unforeseen indications and scope of use. Examples include the utilization of laparoscopy and robotic surgery in pelvic exenteration, lateral pelvic wall lymph node dissection and extra-levator abdominoperineal resections. Many have also appreciated learning new operative approaches and technical skills. Much was also written on the understanding of processes and solutions applied in the healthcare system of host-countries including the patients' journey, healthcare funding, multidisciplinary involvement as well as pre- and postoperative care. Candidates also noted junior doctors' working hours as well as senior surgeon's mentoring style and support for residents and fellows.

For candidates who attended challenging and complex colorectal surgeries relating to inflammatory bowel disease, anal fistulae, pelvic floor and functional bowel disorders, and advanced colorectal malignancies, a consistent message was distilled on the value CTFs as an immersive experience. Many described the benefits of gaining valuable knowledge, learning new surgical techniques, understanding how to negotiate operative frustration points, and refine decision-making processes.

The incentive to travel for the purpose of academic engagement varied considerably. Approximately 75–80 % of fellows attended conferences and were invited as speakers. Many forged subsequent research collaborations and some were involved in departmental scientific activities.

The importance of CTF in the personal and professional journey of surgeons was also highlighted in many reports. Considerable value was stated on the fellow-to-mentor relationship. Some utilized this prize to springboard their career prospects. Others made use of this opportunity to self-reflect and exchange ideas, pursue new friendships and collaborative relationships, and to appreciate different cultures and traditions.

No reciprocal reports were provided by host mentors, centres, or organizations, on the value or impact of TSF/CTF.

## Discussion

The body of evidence supporting specialist fellowship training programmes is considerable; demonstrating significant impact on surgical proficiencies and patient health care outcomes [[28], [29], [30], [31], [32], [33]]. Other research also points towards a consistent increase in the number of applicants pursuing surgical sub-specializations [[34], [35], [36]], matched by an increase in availability of centres offering advanced surgical fellowship training [37]. The factors contributing to these trends are multiple and complex. They include the current structure of graduate surgical training programmes, evolving health care systems and rapid advances in surgical practice. When considering surgical travelling fellowships in general, the picture is mixed. A subset of travelling surgical and colorectal fellowships exhibit a similar structure and framework to graduate training programs. Remaining TSFs, however, vary considerably in duration, structure, experience and level of immersive involvement. For the latter, this level of heterogeneity has perhaps contributed to the lack of scientific evidence evaluating its impact and validity. Nonetheless, general figures published on websites belonging to sponsoring colleges of surgery, surgical associations and societies summarized in Table 1, suggest a pattern of sustained increase in the annual number of fellows from the year 2000 consistent with other specialist training programs.

In this study, we have provided a quantitative analysis on trends of the number of fellows in TSF and CTF and their most sought-after surgical themes in colorectal surgery. We have also provided a qualitative assessment of the values, experiences and personal impacts expressed by travelling fellows from developed and developing countries.

Our results demonstrate a pre-Covid-19 Pandemic showing an increase in the annual number of combined TSF and CTF averaging 26 % since the year 2000. The time-series analysis predicts a continued up-trend beyond the latest available data-points in 2019. The data also shows the distribution of host countries for both TSF and CTF. They are, in order of volume, the USA, Western Europe, The U.K., Australasia, Japan and South Korea.

The incentives to pursue a travelling fellowship, highlighted in this study, are multifactorial. The pursuit of technical surgical knowledge is key. This is clearly demonstrated in the results of our annual distribution of theme analysis, where a correlation was found between the timely emergence, or popularization, of a novel surgical technique, and the year of travel in which it was first recorded as theme-interest. Other incentives include the powerful perception of the added value of TSFs/ CTFs awards, as a privileged opportunity to advance one's own career.

Additional factors include, the opportunity to present completed or ongoing work of research or simply to attend conferences and forums and the ability to connect with master surgeons.

The values distilled in this review are multiple. The most sought-after theme-interests were, consistently: minimally invasive surgery (laparoscopy, robotic surgery and TaTME), the organisation of healthcare, surgical training and mentoring, multidisciplinary management of challenging colorectal cases and new approaches to pelvic floor and proctological conditions. Less quantifiable, but nonetheless frequently expressed were the powerful value of building a lasting mentor-mentee relationship, advancing surgical knowledge, gaining operative insight, embracing the opportunity to reflect and question, the ability to compare and contrast, and finally to represent one's own establishment in a public relation exercise. For international travelling surgical fellows (ITF), their experiences, highlighted in this review reveal a palpable impact on surgeons' attitude, their institutional work-flow in managing complex colorectal cases and on community-based initiatives such as screening programs.

The disruption caused by the COVID-19 pandemic had effectively resulted in the suspension of TSFs/CTFs for two years. Candidates have instead explored the possibilities offered by internet-based platforms. These technologies have enabled borderless and synchronous communications, permitting a broad spectrum of virtual interactions. Substantive evidence has since emerged on their efficacy, validity, cost efficiency and broader reach [[22], [23], [24], [25],^38^,^39^]. It now continues to be adopted by many institutions in mainstream technical and non-technical surgical training [40]. The lasting impact of COVID and these technological advancement on the course of TSF/CTF should be clearer in the following years. Recent advertisements and figures published by the ECP and ASCRS suggest a continued support for CTF.^41,42^ But the future may be shaped by other important factors such as cost and carbon footprint.

Travelling fellowships and international exposure offer numerous advantages, as highlighted in this review. The impact of these experiences on personal and professional development has been demonstrated, emphasizing their significance to candidates. Despite the rise of new technologies and online interactions, these traditional practices are unlikely to be disrupted in the short and intermediate term. However, a hybrid approach has emerged and is expected to continue in the near future, presenting a synergistic solution.

This review is constrained by certain limitations, including its retrospective nature, the heterogeneity and the positively biased nature narrated in post-fellowship reports. To fully realize the potential of Travelling Surgical Fellowships (TSFs) and Colorectal Travelling Fellowships (CTFs), it is imperative to collect prospective standardized feedback. Validating our findings through such feedbacks can inform future guidelines for TSFs, facilitating its standardization and uniformity of its potential.

Furthermore, it is crucial to recognize the limited understanding of the impact of Travelling Surgical Fellowships (TSFs) on inviting host surgeons, institutions, and the surgical community as a whole. Investigating this aspect is imperative to identify the short- and long-term benefits and impacts associated with hosting TSFs. Such research would provide valuable insights and could potentially contribute to the dissemination of these practices and enhance its effectiveness and global reach.

“In an increasingly digital, distance-learning, homogenous, one-size-fits-all era, the James IV Travelling Fellowship captures the essence of this immersive method of exchanging surgical knowledge and keeps it very much alive. There are some things you just can't Google.” [27]

Mr. Euan J Dickson, James IV AS Travelling Fellowship, 2019

## Conclusion

This study provides the first comprehensive review of Colorectal Travelling Fellowships, highlighting their increasing popularity over the past two decades. The results indicate that fellows highly value surgical experiences in areas such as minimally invasive surgery, advanced multimodal management of complex surgical conditions, and interests in health care organisation and surgical education and training. It is evident from our findings that human interaction continues to be essential for fostering collegial relationships and establishing long-term connections among colleagues. To accurately assess the true value of fellowship programs, it is crucial to gather robust data, including value-added fields based on fellows' feedback. Prospective standardized studies are necessary to further explore and validate the outcomes of these programs.

## Author contribution

I am the sole author of this manuscript and have contributed to all aspects of the research, including the design, data collection, analysis, and writing of the manuscript.

## Declaration of competing interest

I declare that there is no conflict of interest regarding the publication of this manuscript.

## Funding statement

I declare that there was no funding received for this research.

## Ethical publication statement

As a literature-based review, this article is exempt.
